# Evaluation of a Non-Chemical Compared to a Non-Chemical Plus Silica Gel Approach to Bed Bug Management

**DOI:** 10.3390/insects11070443

**Published:** 2020-07-14

**Authors:** Salehe Abbar, Changlu Wang, Richard Cooper

**Affiliations:** Department of Entomology, Rutgers, The State University of New Jersey, New Brunswick, NJ 08901, USA; abbar@sebs.rutgers.edu (S.A.); rcooper@sebs.rutgers.edu (R.C.)

**Keywords:** *Cimex lectularius*, silica gel, non-chemical treatment

## Abstract

Bed bug resistance to commonly used pesticide sprays has led to exploring new pesticides and other strategies for bed bug management. Non-chemical methods are effective in bed bug management; however, they do not provide residual protection. Compared to insecticide sprays, dust formulations are considered to provide longer residual control. We evaluated two bed bug management programs in apartment buildings. A building-wide inspection was initially conducted to identify bed bug infested apartments. Selected apartments were divided into two treatment groups: non-chemical plus silica gel dust treatment (10 apartments) and non-chemical treatment (11 apartments). After initial treatment, apartments were re-visited monthly for up to 6 months. During each visit, the total bed bug count per apartment was obtained by examining interceptor traps placed in the apartments and conducting a visual inspection. Mean bed bug count was reduced by 99% and 89% in non-chemical plus silica gel dust and non-chemical treatment, respectively. Non-chemical plus silica gel dust treatment caused significantly higher bed bug count reduction than the non-chemical treatment at 6 months. Bed bugs were eradicated from 40% and 36% of apartments treated with non-chemical plus silica gel dust treatment and non-chemical treatment, respectively.

## 1. Introduction

The common bed bug, *Cimex lectularius* L. (Hemiptera: Cimicidae), has recently resurged in the U.S. The impacts from bed bug infestations on people’s lives include physical, medical, and economic impacts [1,2,3,4]. Low-income communities suffer high bed bug infestation rates [5,6,7,8], due to the failure of residents to recognize or report the presence of bed bugs, inability or refusal of residents to comply with requests for cooperation during the control process, and low-quality pest management service [9,10,11,12]. Chronic infestations often cause huge financial losses for the property management, and serve as a source of new infestations. Therefore, property managers must adopt more effective bed bug detection and control strategies [8,11]. 

Despite many control approaches developed for the pest management industry, insecticide application, which is often economical and convenient, remains the most popular bed bug control method among professionals [13,14]. Pesticide application can result in human pesticide exposure and increases bed bug resistance [15,16,17]. Widespread reports on pyrethroid resistance from different countries have accelerated the development and registration of newer formulations and chemistries for effective bed bug management. Insecticide dust is used for bed bug control offering better residual protection than pesticide sprays [18,19]. Silicon dioxide-based (SiO_2_) desiccant dusts are popular, because of their low mammalian toxicity, long residual life (in dry environments), and effectiveness against pyrethroid-resistant bed bugs. When bed bugs were exposed to CimeXa insecticide dust during a choice bioassay and a forced-exposure bioassay, 95% mortality was achieved after 1 day, and 100% mortality was observed after 5 and 10 days [20]. In another laboratory study, CimeXa insecticide dust at the label rate produced 100% mortality in highly pyrethroid-resistant and insecticide-susceptible strains of bed bugs within 3–4 days [21]. Ninety-eight percent mortality was achieved after 1 day when silica gel dust, in a water solution, was applied to bed bugs that were moderately resistant to pyrethroids [22]. Forced exposure to silica gel dust, at a dose of 1.34 mg/cm^2^, caused > 95% bed bug mortality within 1 day [23]. Forced exposure of bed bugs to silica gel dust for just 1 min 14 s was enough to cause 100% mortality [24]. Drione (40% SiO_2_, 1% pyrethrin, 10% piperonyl butoxide) produced 100% mortality in two highly resistant, one moderately resistant, and one susceptible stain of bed bugs within 72 h in a laboratory study [25]. The application of Syloid (99.6% SiO_2_) combined with CO_2_, as a bed bug stimulant, resulted in 100% bed bug mortality in 5 and 2 days with low (0.1 g/m^2^) and high (3 g/m^2^) concentrations in the laboratory [26]. In a recent field study, application of 28 to 85 g/apartment of CimeXa dust alone in six apartments resulted in 82 and 98% reduction in average bed bug counts after 1 and 12 weeks, respectively [23]. 

Non-chemical methods, such as proper inspection, frequent laundering, de-cluttering, vacuuming, encasement of mattresses and box springs, steam treatment, installation of passive pitfall-style traps (interceptors), discarding severely infested items, and structural heat treatment, are all effective techniques; however, they do not provide residual protection [27,28,29]. Cooper et al. (2016) evaluated an integrated pest management (IPM) program in a low-income housing community over a 12-month period [12]. The program included the education of residents and staff, and the implementation of a combination of monitors, steam, encasement, de-cluttering, laundering, and limited insecticide applications. Results showed the number of treatment visits required to eliminate an infestation is correlated with infestation levels, and moderate to severe infestations can be eliminated without using large amounts of pesticides. The IPM program resulted in a 98% reduction in bed bug counts among treated apartments and reduced infestation rates in the building from 15 to 2.2% after 12 months. Cooper et al. (2016) also showed mass trapping alone eliminated 50% of low-level infestations after 16 weeks [30]. Eradication efforts often require numerous service visits from a pest management professional, and involve the use of a variety of chemical and non-chemical control measures, along with the selective treatment or disposal of infested furniture and other personal belongings [31,32,33].

Although silica gel dust is a very promising insecticide based on preliminary studies, extensive data on its effectiveness under field conditions is lacking. We designed and implemented two programs for bed bug management in affordable housing communities for elderly and disabled residents in Trenton and Linden, New Jersey. The programs included initial building-wide inspection to identify infestations, implementing a combination of non-chemical methods and silica gel dust, and non-chemical methods alone. Our primary objective was to determine whether non-chemical plus application of silica gel dust would result in a faster and a higher rate of bed bug eradication, compared to non-chemical control in apartments.

## 2. Materials and Methods 

### 2.1. Study Sites

The study was conducted in two high-rise apartment buildings in Trenton and one high-rise apartment building in Linden, New Jersey. At Trenton, the two apartment buildings consisted of a total of 246 and 229 apartments, respectively, and were managed by a private company. At Linden, the building consisted of a total of 201 apartments, and was managed by a public housing authority. These included one-bedroom apartments or studios. Each apartment was occupied by one or two senior residents (>62 years old) during the period of the study. Both housing communities had bed bug infestations for 7 years, prior to this study. The number and type of methods used by residents to combat bed bugs in their apartments varied widely from one apartment to the next. Methods used by residents included applying insecticides, discarding infested furniture and other personal belongings, and installing encasements to the mattresses and box springs or keeping the original plastic cover on mattresses and/or box springs. Pesticides containing pyrethroids were applied by contracted pest management professionals at Trenton once the bed bug infestation was reported. A combination of pesticides application and installing bed bug interceptors was used by licensed housing staff at Linden to treat infestations. Pesticides containing diatomaceous earth dust, pyrethroids, and pyrethroid-neonicotinoid mixtures were utilized at Linden.

### 2.2. Selection of Apartments

A building-wide inspection was initially conducted in March 2018 at Trenton and in April 2019 at Linden to identify bed bug infested apartments. Approval from Rutgers University Institutional Review Board (Pro20170001957) was obtained prior to the study. Climbup Insect Interceptors^®^ (Susan McKnight, Inc., Memphis, TN, USA), hereafter referred to as interceptors, were installed under the legs of beds and upholstered furniture in apartments with live bed bugs or signs of bed bug infestation, and were inspected after 11–14 d placement. An average of six interceptors (range: 4–8) were installed in each apartment. The total bed bug count from the interceptors laid per apartment was adjusted by dividing the counts by the number of days of placement then multiplied by 14 to yield 14 d interceptor counts. At Trenton, the two buildings had infestation rates of 8.4% and 5.5%, respectively. At Linden, 18.2% of the apartments were found to be infested in the building. We selected 21 apartments for this study (10 from Trenton, and 11 from Linden). The selection was based on residents agreeing to participate in the study and at least 10 bed bugs detected through visual inspection and interceptor counts. In each building, selected apartments were divided into two treatment groups. In total, there were 10 apartments in the non-chemical plus silica gel dust treatment group, and 11 apartments in the non-chemical treatment group. 

### 2.3. Treatment Methods

#### 2.3.1. Initial Treatment

Within 4 weeks following the initial inspection of the apartments, the residents of selected apartments were asked to stop using any insecticides. We did not ask residents to do extensive preparations typical of those required by most pest management companies, such as removing items around the beds and sofas, bagging bed linens, removing mattresses and box springs, or emptying closets and drawers. Such requests for extensive preparation have been shown to be unnecessary [12]. However, residents were asked to cooperate with specific requests made following the initial treatment and during follow-up visits. On the initial treatment day, a visual inspection was conducted immediately before treatment. The number of bed bugs observed during visual inspection and in interceptors prior to the treatment were used to calculate total bed bug count per apartment. In the non-chemical treatment, the following techniques were used during initial treatment:

(1) Infested furniture was steamed (The Steamax, Amerivap Systems, Dawsonville, GA, USA) and/or vacuumed (Atrix, Model VACBP1, Atrix International, Burnsville, MN, USA). In some cases, bed bugs were hand-removed with forceps;
(2) Mattresses and box springs were encased with vinyl plastic encasements (Bedding Essentials, PA, USA);
(3) Interceptors were installed under the legs of beds, upholstered furniture, and other infested areas. Based on our initial visits and detection of infested areas in each apartment, additional interceptors were installed in areas away from resting and sleeping areas in nine apartments, to capture bed bugs in the areas with known bed bug activity. The additional interceptors were part of non-chemical treatment in this study. The interceptors installed in resting and sleeping areas were considered as bed bug monitors. An average of eight interceptors (range: 4–13) were installed in each apartment;
(4) Bed linens and pillow covers were bagged in plastic bags and a note left on the bag instructing the resident to hot launder the bagged items. We also asked residents to reduce clutter around beds and upholstered furniture;
(5) Residents were provided with a pamphlet, listing methods to prevent bed bug infestations and steps they could take to help eliminate bed bugs in their apartments.

In the non-chemical plus silica gel dust treatment, in addition to non-chemical techniques, CimeXa dust (92.1% amorphous silica, 6.1 g/m^2^, Rockwell Labs Ltd, North Kansas City, MO, USA) was applied using an Exacticide power duster^®^ (Technicide, Herber City, UT, USA) to bedroom and living room furniture, as well as the baseboards in the bedroom and living room. CimeXa dust applications were made, in accordance with label directions.

#### 2.3.2. Follow-Up Visits

Follow-up visits were conducted at 0.5, 1, 2, 3, 4, 5, and 6 months, unless the infestation was deemed eliminated prior to 6 months. An infestation was deemed eliminated if no bed bug activity was observed over three consecutive visits in interceptors and the visual inspection of beds and upholstered furniture, and there were no new reports of bed bug activity or bite symptoms by the resident. During each follow-up visit, a thorough visual inspection was conducted to identify live bed bugs. Interceptors were also inspected for bed bug activity (live or dead). The total bed bug count per apartment at each visit was calculated by adding the interceptor count and the visual inspection count. Bed bugs found during visual inspections were physically removed or destroyed through vacuuming, steaming or removal by hand. Bed bugs in interceptors were removed and flushed down the toilet. In the non-chemical group, treatment methods were the same as those used during the initial service except for installing mattress and box spring encasements. In the non-chemical plus dust group, in addition to the non-chemical methods, silica gel dust was reapplied in areas of continued bed bug activity, according to the label directions. In both treatment groups, encasements were inspected for bed bug activity, as well as for rips and tears. Any live bed bugs observed were physically removed and any rips or tears were either repaired by patching tears with duct tape, or replacing the encasement if torn badly. During follow-up visits, no additional treatment was carried out when fewer than five dead bed bugs were detected. This decision was made based on previous studies showing a low level of bed bug infestations can be eliminated without treatment [30]. Apartments were not treated at the last visit at 6 months. Property management was made aware of any apartments that still had bed bug activity at the conclusion of the study, so they could arrange for additional servicing of the apartments. Treatment tasks were carried out by Rutgers University researchers. Three Cooper Pest Solution technicians participated in the initial treatment at the Trenton site. Two or three researchers serviced each apartment during each visit. The time spent in each serviced apartment was recorded during every visit.

### 2.4. Statistical Analysis

One-way analysis of variance (ANOVA) was used to compare initial bed bug counts, initial and total service time, and the number of retreats. Tukey’s HSD (honestly significant difference) test was used to separate the means. Changes in the logarithmically transformed bed bug count data over the observation period were analyzed using mixed model (Proc Mix in SAS software). Fisher’s exact test was used to compare the effectiveness of the two treatments by evaluating the proportion of apartments with ≤1 bed bugs at 6 months. Kruskal-Wallis test was used to compare the median number of visits between treatments. The bed bug count over the initial 2 weeks before treatment was doubled, to justify the count for 0 month, and to generate bed bug counts with the same time intervals from month 0–6. The number of bed bug counts from interceptors for month 0.5 was added to the counts for 1 month, to generate bed bug counts with the same time intervals from month 0–6. In nine apartments, additional interceptors in areas away from sleeping and resting area were included in the trap counts. Among these, only in four apartments (two in each treatment group) did using additional interceptors result in higher bed bugs counts. In other apartments, with additional interceptors, zero bud bugs were counted in additional interceptors. Bed bug counts from addition interceptors at 1 month were added to the count for 0 month, to compensate for the lower number of interceptors during the initial visit. All analyses were conducted using SAS software 9.4 [34].

## 3. Results

**Figure 1:**
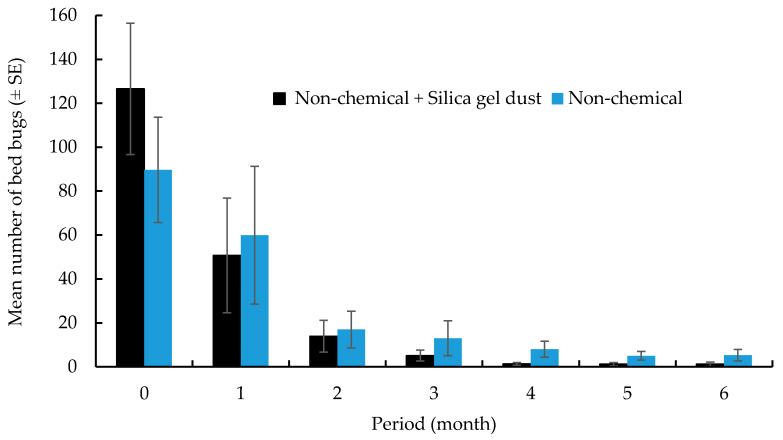
Changes in bed bug count after treatment. The bed bug count was based on a combination of interceptor count over 1 month period and live bed bugs found during visual inspection at the sampling point.

Details of the bed bug counts in each apartment are listed in Table 1. The initial mean bed bug counts in non-chemical plus silica gel dust and non-chemical treatment groups was 127 ± 31 and 90 ± 25, respectively. They were not significantly different (F = 1.1; df = 1, 19; *p* = 0.31). At 6 months after the initial treatment, the bed bug count was reduced by 99 ± 1% and 89 ± 6% in non-chemical plus silica gel dust and non-chemical treatments, respectively. They were significantly different (t = 2.15, df = 94, *p* = 0.03) (Figure 1). The bed bug count distribution at 6 months is shown in Table 2. At 6 months, 90% of apartments from the non-chemical plus silica gel dust group had zero to no more than one bed bug, while only 46% of apartments had zero to no more than one bed bug in the non-chemical group. Fisher’s exact test showed non-chemical plus silica gel dust had significantly higher proportion of apartments with ≤1 bed bugs than the non-chemical treatments (χ^2^ = 4.68, df = 1, *p* = 0.04). The elimination of bed bugs (zero counts in three consecutive visits) was only achieved in 40 and 36% of apartments treated with non-chemical plus silica gel dust and non-chemical methods, respectively. 

The median (min, max) number of visits required to achieve at least one bed bug count of zero were 5 (2, 7), and 4 (2, 5) visits, in non-chemical plus silica gel dust and non-chemical treatments, respectively. There was no significant difference in the mean number of follow-up treatments (χ^2^ = 0.17, df = 1, *p* = 1.86), and total number of visits (χ^2^ = 0.07, df = 1, *p* = 0.79) between the two treatment groups. The mean initial treatment time per apartment was not significantly different between the treatment groups (F = 2.3; df = 1, 19; *p* = 0.17) (Table 3). Similarly, there was no significant difference in the total treatment time per apartment between treatment groups (F = 0.44, df = 1, 18, *p* = 0.52). In the 10 apartments treated with non-chemical plus silica gel dust method, average of 7.9 g/apartment silica gel dust was used during the entire study period.

## 4. Discussion

This is the first study to compare the effectiveness of silica gel dust combined with non-chemical methods to non-chemical methods only, for the control of bed bugs in the field. Reduction of bed bug count was significantly different between treatments at 6 months. A greater proportion of apartments had a count of 1 or fewer bed bugs at 6 months in the non-chemical plus silica gel dust group. At 6 months, only one apartment in the non-chemical plus silica gel dust group had a bed bug count greater than one, and none had a count greater than 10. In comparison, in the non-chemical only group, six apartments still had bed bug counts greater than one, with two of these apartments having greater than 20 bed bugs. Bed bug counts were also reduced to very low levels over a shorter period in the non-chemical plus dust, compared to the non-chemical only group. Among the infestations not eliminated in the non-chemical plus dust group, only one apartment had a count of greater than five bed bugs between months 4–6. In comparison among those not eliminated in the non-chemical only group, 57% of apartments still had bed bug counts greater than 10 over the same 4–6-month period. The difference in bed bug count distribution between the two treatment groups is of importance in the applied setting based upon the findings of Cooper et al., (2016) [30] which demonstrated that apartments with low-level populations often decline to zero without chemical intervention. Thus, presence of one bed bug in an apartment does not necessarily imply the rebound of the population in the future. 

The results of this study are comparative to bed bug reduction and elimination rates reported in other studies that used a combination of non-chemical and chemical methods or non-chemical methods only. For instance, a combination of non-chemical and Alpine dust (0.25% dinotefuran, 95% diatomaceous earth dust) resulted in 97% bed bug reduction and bed bug elimination in 30% of treated apartments [11]. Similarly, Wang et al. (2009) [31] reported a 98% bed bug reduction and 50% bed bug elimination in apartments treated with diatomaceous earth dust and non-chemical methods at 10 weeks. A bed bug count reduction of 99.9%, and bed bug elimination of 44% were achieved in apartments treated with non-chemical treatment and 0.075% Temprid SC (imidacloprid and cyfluthrin), Tempo dust (1% cyfluthrin) or diatomaceous earth dust [32]. A combination of Tempo dust, Alpine aerosol, and non-chemical methods caused 25% bed bug elimination, and bed bug count reduced 92% at 12 weeks [33]. Implementing an IPM program in a building-wide study reduced bed bug count of 98% was reported by Cooper et al., 2016 [12]. Likewise, in another field study, a 98.6% reduction in bed bug count and 67% bed bug elimination were achieved at 10 weeks in apartments treated with a non-chemical treatment protocol [32].

This and previous studies showed very high reduction in bed bug numbers after implementing a thorough treatment program, although bed bugs were not eliminated in all treated apartments. In most apartments, bed bug elimination can be achieved with limited resident cooperation. However, cooperation by residents is helpful, and aids in the speed of elimination in some apartments. In the non-chemical plus silica gel dust group, two apartments had challenging conditions. The first apartment was extremely cluttered, and the resident did not cooperate with laundering the bagged items and de-cluttering. The infested items were reused and scattered adjacent to the mattress and box spring. There was no bed frame to support the box spring and mattress. Bed bugs were observed in the traps around the sleeping area at each visit. The infestation was reduced by 98% in 4 months, and reduced to zero at 5 months, after we had the opportunity to communicate with the resident to clarify the importance of the hot laundering of infested items, and to avoid using them before they are disinfested. In the second apartment, the bed bug count was reduced by 98% at 4 months, however, the resident refused to acknowledge that the apartment was infested with bed bugs at 4 months, and refused to permit further treatment. Complex wicker furniture infested with bed bugs in the apartment made it difficult to treat effectively. Additionally, the resident removed the encasement from the air mattress after initial treatment, and refused to keep the mattress encased. Ten bed bugs were found in this apartment during the last inspection. 

In the non-chemical group, resident cooperation was lacking in three apartments where significant obstacles to control existed. In two of these apartments, badly infested furniture (one recliner, sofa, or bed frame) was the primary infestation location. The furniture in each of the apartments was complex or in disrepair, provided many hiding places for bed bugs, and was difficult to treat by steamer or vacuum. In each apartment the furniture was steamed 7–8 times during the study period. In one of these apartments 246 first instar bed bug nymphs were counted in the interceptors during the first follow-up visit. It seemed that some bed bug eggs were laid and hidden inside the complex furniture and far from steam and vacuum treatments. The residents of these apartments were not inclined to remove the hard-to-treat furniture from their apartments. The bed bug count reduced by 50% and 98% at 6 months in these two apartments. The third apartment was heavily cluttered. The resident never assisted with hot laundering the infested bagged items and linens. These items were reused and scattered around the apartment before every treatment. These cases demonstrate that, even with very careful inspections and treatments, a small portion of the apartments can still present challenges to control. The bed bug count reduced to 48% at 6 months in this apartment. While infestations can be reduced significantly, in some cases elimination is not possible without cooperation from residents. The finding is consistent with that reported by other studies [11,30,32].

Other factors contributed to the length of time to eliminate or reduce infestations to low levels. More frequent visits might have produced a more rapid and higher rate of elimination. Biweekly inspection and treatment have been conducted in previous studies, and resulted in a higher bed bug elimination rates [4,30]. However, biweekly visits are more labor-intensive and time-consuming. It is possible that the quantity of silica gel dust applied in this study was insufficient to ensure high efficacy under field conditions. We applied about 8 g silica gel dust per apartment, while 28 to 85 g of this product was used in a field study and resulted in 98% bed bug reduction after 4 months [23]. Another important factor that likely contributed to our elimination rates is the robust methodology used to determine when an infestation was considered eliminated. Criteria used to determine bed bug elimination in this study is much more challenging, compared to methods used by the pest management industry. Our study required three consecutive months of zero bed bugs using a combination of visual inspection and interceptor trap counts. Typically, pest management professionals discontinue service following a single visit without bed bug activity detected through visual inspection alone. In our study, among apartments where elimination was not achieved, bed bug activity was detected in interceptors, but not through visual inspection, in 70% of the apartments. This is consistent with another field study that showed that following treatment, the likelihood of capturing bed bugs away from sleeping and resting areas is about 2.6 times more than at sleeping and resting areas [30]. The criteria of this study suggest that the pest management industry should utilize more than visual inspection alone to determine when bed bug treatment should terminate. Using a less robust protocol, elimination rates would have been much higher for the non-chemical plus silica gel dust group, compared to the non-chemical only group. Among those not eliminated, 80% of the apartments in the non-chemical plus silica gel dust group had a count of zero during one or more visits between months 4–6. In comparison, in the non-chemical only group, among those not eliminated only 28% of the apartments had a count of zero between months 4–6.

The lack of a significant difference in the initial treatment time between treatments was probably a result of all apartments being carefully inspected immediately before each treatment. In this study, we included heavily cluttered apartments. Silica gel dust application took a very short amount of time. The inspection was necessary for identifying the harborages and determining where to apply the treatments. Comprehensive pre-treatment preparation is time-consuming and labor-intensive, and not necessary for eliminating most of the bed bug infestations [12]. Most professional pest management companies require residents to prepare an extensive preparation list [35]. We preferred to not require any preparations from the residents prior to each treatment visit. Preparations were not feasible for senior or disabled residents and may complicate control by dispersing bed bugs. Not requiring preparation by residents factored into the length of the treatment. We inspected clothes, bed sheets, and any other clutter surrounding the beds and furniture before bagging infested items.

## 5. Conclusions

The results of this study have important implications in the implementation of bed bug management programs in multifamily housing communities. These include: (1) the non-chemical plus silica gel dust method can be more effective in reducing bed bug numbers, compared with the non-chemical only method; (2) the complexity of the infested furniture and the clutter surrounding the sleeping and resting areas can create refuges for bed bugs, and subsequently, delay the elimination by months; (3) the lack of resident cooperation with bed bug control procedures contributed to elimination failure in a few cases. Applying these findings in development of future bed bug management programs will help eliminate bed bugs more safely and efficiently.

## Figures and Tables

**Table 1:** Bed bug counts based on combination of visual inspection and interceptor trap counts.

Location	Apt. No.	Treatment	Number of Bed Bugs Recorded from Monthly Visit						
			0 Month	1 Month	2 Month	3 Month	4 Month	5 Month	6 Month
Linden	1	Non-chemical plus silica gel dust	155	31	4	3	1	1	0
Linden	2	Non-chemical plus silica gel dust	23	6	1	2	0	0	0
Linden	3	Non-chemical plus silica gel dust	29	3	0	2	1	2	1
Linden	4	Non-chemical plus silica gel dust	88	56	45	2	1	1	0
Linden	5	Non-chemical plus silica gel dust	88	36	1	0	0	0	0
Linden	6	Non-chemical plus silica gel dust	206	37	7	2	0	0	1
Trenton-Bld.1	7	Non-chemical plus silica gel dust	53	0	0	0	0	0	0
Trenton-Bld.1	8	Non-chemical plus silica gel dust	60	0	0	0	0	0	0
Trenton-Bld.2	9	Non-chemical plus silica gel dust	291	38	11	25	4	0	0
Trenton-Bld.2	10	Non-chemical plus silica gel dust	272	291	70	15	6	8	10
Linden	1	Non-chemical	224	114	15	5	2	1	3
Linden	2	Non-chemical	18	0	0	0	0	0	0
Linden	3	Non-chemical	45	4	17	12	37	15	23
Linden	4	Non-chemical	48	13	10	5	26	13	24
Linden	5	Non-chemical	220	364	96	94	9	18	3
Trenton-Bld.1	6	Non-chemical	203	95	7	2	0	0	0
Trenton-Bld.1	7	Non-chemical	52	33	41	20	14	6	2
Trenton-Bld.1	8	Non-chemical	82	2	0	0	0	0	0
Trenton-Bld.2	9	Non-chemical	52	11	0	1	0	1	2
Trenton-Bld.2	10	Non-chemical	16	10	1	4	0	1	1
Trenton-Bld.2	11	Non-chemical	26	4	0	0	0	0	0

**Table 2:** Effect of two treatment programs on bed bug count reduction at 6 months.

Treatment	% Apartments with Zero Bed Bugs	% Apartments with 1 Bed Bug	% Apartments with ≥2 Bed Bugs
Non-chemical plus silica gel dust	70	20	10
Non-chemical	36	9	55

**Table 3:** Treatment information of the infested apartments.

		Mean (± SE)				
Treatment	n	Dust Usage per Apartment (g)	Initial Treatment Time per Apartment (min)	Total Treatment Time per Apartment (min)	Number of Vacuum Treatments	Number of Steam Treatments
Non-chemical plus silica gel dust	10	7.9 ± 0.2	51 ± 6	144 ± 12	0.7 ± 0.2	1.9 ± 0.4
Non-chemical	11	-	42 ± 3	161 ± 21	1.0 ± 0.2	4.5 ± 0.7
